# Attitudes toward rehabilitating inmates among occupational therapy students in the United States

**DOI:** 10.3352/jeehp.2019.16.6

**Published:** 2019-03-25

**Authors:** Sarah Catherine Tucker, Hon Keung Yuen

**Affiliations:** Department of Occupational Therapy, School of Health Professions, University of Alabama at Birmingham, Birmingham, AL, USA; Hallym University, Korea

**Keywords:** Attitude of health personnel, Occupational therapy, Students, Health professions, Criminals, United States

## Abstract

**Purpose:**

This study was to examine occupational therapy (OT) students’ attitudes toward rehabilitating inmates and validate an instrument used to measure their attitudes.

**Methods:**

OT students (n=128) from one university in Alabama, United States, completed an online survey exploring their attitudes toward rehabilitating inmates, which was assessed using the Rehabilitation Orientation Scale (ROS), a 7-point scale. Dimensional structure, internal consistency reliability, construct validity, and relations to other variables of the ROS was evaluated using factor analyses, Cronbach’s alpha, known-groups method, and univariable correlations, respectively.

**Results:**

Unidimensionality of the ROS was confirmed with an alpha coefficient of 0.90. The mean ROS score of the respondents was 5.1; a score toward 7 indicated a more supportive attitude. About 60% of the respondents reported supportive attitudes (i.e., an ROS score ≥5). Respondents’ ROS scores were significantly higher than those of the public and criminal justice professionals. Female students reported a more supportive attitude than males. Multiple regression analysis indicated that respondents’ consideration of working in prison settings after graduation and their perception that OT has a role in prison settings were significantly associated with support for rehabilitating inmates, after controlling for gender and an acquaintance with someone who has been incarcerated.

**Conclusion:**

Results indicated that the ROS demonstrated adequate psychometric properties as it applied to this population. The majority of respondents reported supportive attitudes toward rehabilitating inmates. Consideration of working in prison settings after graduation and the perception that OT has a role in prison settings were 2 independent factors associated with respondents’ attitudes toward rehabilitating inmates.

## Introduction

With the recent change in the US government policy to reform the federal prison system, the First Step Act was effective on Dec 21, 2018 as signed by President Donald Trump. This Act will allow early release of federal inmates from prisons, improve treatment and rehabilitation of inmates to help them transition back to the community, and expand a wide range of community-based re-entry programs for ex-offenders [1]. Consequently, more health professionals will be needed to work with inmates and ex-offenders to achieve this national rehabilitation goal. Occupational therapists play an important role in the rehabilitation of inmates in correctional and community settings [2]. Their attitudes toward rehabilitating inmates may affect the therapeutic process, quality of rehabilitation services, and treatment outcomes [3].

To better understand attitudes toward rehabilitating inmates among occupational therapy (OT) students is critical to assist educators in the preparation of graduates to work with this growing population. This study was, therefore, to examine OT students’ attitudes toward rehabilitating inmates. To reach this goal, we also sought to validate an instrument used to measure rehabilitation orientation toward inmates.

## Methods

### Ethical statement

The Institutional Review Board of the University of Alabama at Birmingham (UAB) approved the study protocol (150708008).

### Study design

A cross-sectional descriptive design was used.

### Subjects

A cover letter explaining the purpose of the study with the survey instrument Uniform Resource Locator was e-mailed to all 151 OT graduate students of the 3 years (1st year=57 students, 2nd year=54, and 3rd year=40) at UAB in early September 2015. The survey questions were posted on SurveyMonkey. Data were collected through late November 2015, with a follow-up reminder e-mail sent in early October. Two university OT faculty, one with expertise in research methodology and statistics, and the other with 6 years of volunteer experience in a prison setting designed the survey questions.

The survey with 9 questions was intended to collect data that could be used to identify factors associated with OT students’ attitudes toward rehabilitating inmates, which was measured by the Rehabilitation Orientation Scale (ROS) [4]. In addition to demographic information (age, gender, and race) and current year in the OT program, students were asked about their consideration of working in prison settings after graduation, whether they knew someone who has been incarcerated (no=0, or yes=1), whether they had exposure to a therapist working in the prison setting (no=0, or yes=1), and opinion about whether or not OT has a role in prison settings (yes, not sure, or no). We recoded the “not sure” response to “no” for the sake of easier interpretation (no=0, or yes=1) in the data analysis process. The response to the question on consideration of working in prison settings after graduation was anchored by 2 verbal qualifiers, highly unlikely=1 and highly likely=6, on a 6-point Likert-type scale.

The ROS consisted of 9 items with responses anchored by 2 verbal qualifiers, very strongly agree=1 and very strongly disagree=7, to indicate levels of agreement with each item. Four of the items were worded supportively in attitudes toward rehabilitating inmates, and responses were re-coded in order to attain consistency when creating the composite scale score. The ROS, which demonstrated satisfactory psychometric properties, has been used to measure attitudes toward rehabilitation and punishment of inmates among staff working in prison settings [4]. However, there were no prior studies to evaluate the internal structure of this instrument and ROS has not been validated for rehabilitation professional students.

### Analytic methods

Factor analyses and item analysis were conducted to evaluate the internal structure of the ROS. It was recommended that formation of the composite score of a measure is conducted only when its internal structure is unidimensional [5]. Exploratory factor analysis (EFA) using the principal axis factoring method was conducted to evaluate the internal structure of the ROS. Number of factors extracted was determined by examining the Cattell’s scree plot, and Horn’s parallel analysis. In Horn’s parallel analysis, eigenvalues from the factor solution of the ROS score were compared to eigenvalues from a randomly generated data matrix of the sample (i.e., 9 items×128 respondents). Number of factors retained for the ROS was determined by the eigenvalues larger than those of the randomly generated data.

Confirmatory factor analysis was performed to validate the factor model of the ROS. Factor loadings, model fit statistics, and modification indices were examined to determine whether the factor model of ROS provided an acceptable fit to the observed data. Goodness-of-fit statistics of the confirmatory factor analysis (CFA) model was evaluated using the comparative fit index (CFI), non-normed fit index (NNFI), root mean square error of approximation (RMSEA), and standardized root mean square residual (SRMR). In general, CFI ≥0.95, NNFI ≥0.90, RMSEA ≤0.08, and SRMR ≤0.08 are indicative of good fit with acceptable CFA models [6]. CFA was conducted using the LISREL ver. 9.2. software program (Scientific Software International Inc., Lincolnwood, IL, USA).

Internal consistency reliability of the ROS was estimated using Cronbach’s alpha. Bivariable association (Pearson product-moment correlation, Spearman rank-order correlation, or point-biserial correlation) between variables in the survey and the ROS score was conducted, depending on the level of measurement of the variables. Known-groups method was used to compare the ROS score of the respondents with that of the historical comparison group using an independent sample t-test. Specifically, ROS scores from the public and criminal justice professionals were used for comparison [7].

To identify variables that would influence the respondents’ attitudes toward rehabilitating inmates, we used multivariable linear regression analysis. For the preliminary analysis related to the multivariable linear regression modeling, explanatory variables were initially screened for consideration in the model using bivariable association between each explanatory variable and the response variable, which was the ROS score.

For the adjusted analysis, we fit a multivariable linear regression model with the ROS score as the response variable. We considered explanatory variables as candidates for inclusion in the multivariable linear regression analysis if they were significantly associated with the ROS score (P<0.10) in the bivariable analyses. Backward elimination procedure, supplemented with best subsets method, was used to obtain the most parsimonious set of explanatory variables for the respondents’ ROS scores. The Automatic Linear Modeling used the best subsets method to compute the statistical relationship between all possible combinations of the explanatory variables and the ROS, and compared several model selection criteria across all models to determine which model best fit the observed data. Explanatory variables whose regression coefficients had P-values <0.05 were retained in the multivariable regression model. EFA, bivariable association, and multiple regression analysis were conducted using IBM SPSS for Windows ver. 23.0 (IBM Corp., Armonk, NY, USA).

## Results

We received 128 completed surveys, with an estimated response rate of 84.8%. The mean and standard deviation (SD) age of the students were 24.2±3.5 years old (range, 20 to 45 years). The mean and SD of respondents’ consideration of working in prison systems after graduation was 3.1±1.5, with 39.1% of respondents reported indicating the consideration of working in prison settings after graduation as likely (i.e., a score of 4 or above in the 6-point scale). The raw data are available in Supplement 1.

### Internal structure of the Rehabilitation Orientation Scale

An examination of the scree plot revealed a clear break after the first factor, therefore, retaining one factor was appropriate for the data. Horn’s parallel analysis confirmed the findings as there were only one factor with eigenvalues exceeding those of the randomly generated data. The factor of the ROS accounted for 56.8% of the total variance (eigenvalue=5.11). The factor loading of the 9 items was all above 0.55, ranging from 0.57 to 0.86, indicating that all items were considered to be important [8]. Results of the CFA suggested a reasonably good fit between the observed data and the hypothesized one-factor model, with CFI=0.96, NNFI=0.94, RMSEA=0.09, and SRMR=0.05.

After the recoding, the 9 items of the ROS were summed to form a composite, with a higher score indicating a more supportive attitude toward rehabilitating inmates (theoretical range, 9 to 63). The mean and SD of the ROS composite score was 5.06±1.17, with item means ranging from 4.05 to 5.49. The majority of the respondents (58.6%) reported a mean score of ROS of at least 5 (supportive attitudes toward rehabilitating inmates), 25% reported neutral or showed a tendency toward supportive attitudes (i.e., scores between 4 and 5), and 16.4% reported non-supportive attitudes (i.e., a score below 4). Table 1 shows the frequency, mean and SD scores of each of the 9 ROS items rated by the respondents. Cronbach’s alpha coefficient of the 9 ROS items was 0.90 (95% confidence interval, 0.88 to 0.93), which were considered to be excellent [9]. Item analysis indicated that eliminating any one item would not increase the alpha coefficient. The corrected item-to-total correlations between scores of an individual item and the ROS composite score of the remaining items were all ≥0.55 (moderate correlation), ranging from 0.55 to 0.81.

Known-groups method revealed a significant difference in ROS scores was observed (P<0.001) between the present and study samples of Cullen et al. [7], with the respondents in the present study scoring significantly higher in the ROS (i.e., holding a more supportive attitude toward rehabilitating inmates). The mean and SD ROS scores of the participants (the public and criminal justice professionals, n=430) in the survey of Cullen et al. [7] were 4.12±0.86.

### Factors associated with the respondents’ rehabilitation orientation toward inmates

From the results of the bivariable analyses, variables with a P-value <0.10 included in the multivariable linear regression model were: consideration of working in prison settings after graduation, perceived OT has a role in prison settings, had exposure to a therapist working in the prison setting, knowing someone who has been incarcerated, and gender with female students demonstrating a more positive attitudes toward rehabilitating inmates than males (Table 2). The final model derived from the backward elimination procedure included 4 variables (consideration of working in prison settings after graduation, perceived OT has a role in prison settings, knowing someone who has been incarcerated, and gender). The model was supported by the best subsets regression analysis. The multivariable linear regression model with the 4 explanatory variables produced R^2^=0.19, adjusted R^2^ =0.17; F (4, 123)=7.36, P<0.001, with 19% of the variability of ROS scores was explained by these 4 explanatory variables. The Mallows’ Cp values was 5.1. The recommended Mallows Cp value for a model with 4 predictors was 5, which indicated minimal bias in this model. However, in this model, only consideration of working in prison settings after graduation, and perceived OT has a role in prison settings were significantly associated with the ROS score. The coefficient of each explanatory variable with significant effect on the respondents’ ROS score is shown in Table 3.

## Discussion

The majority of respondents demonstrated strongly supportive attitudes toward rehabilitating inmates. The results revealed the internal structure of the ROS was consistent with the one-factor structure described in the original study [4]. Evidence of reliability and validity was also shown based on Cronbach’s alpha, known-groups method, and relations to other variables. The findings were consistent with previous studies that university students (medical, nursing, psychology, and criminology) hold a stronger supportive attitude toward rehabilitating inmates than the general public and correctional officers and law enforcement officers [10-12]. Female students demonstrated a more positive attitude toward rehabilitating inmates than males [10]. Multivariable linear regression analyses indicated that respondents’ consideration of working in prison settings after graduation and perceiving that OT has a role in prison settings were significantly associated with support for rehabilitating inmates after controlling for gender and knowing someone who has been incarcerated.

Individuals who choose to become an occupational therapist are predisposed to help individuals to recover from illness, and this trait is then fostered in their academic and clinical training [13]. To be in the OT program may further shape the respondents’ attitudes and beliefs in the efficacy of treatment and supports for inmate rehabilitation, as this is the philosophy of their profession [14]. However, this phenomenon was not supported in the present study. There was no significant difference in the respondents’ attitudes toward rehabilitating inmates across the first year, second year, and third year cohorts surveyed. This may attribute to no further emphasis on the roles of OT in prison setting after the first year of the curriculum in the UAB academic program.

Even though about 60% of the student respondents reported supportive attitudes toward rehabilitating inmates, as educators, we should pay attention to the 41.4% of student respondents who showed less or non-supportive attitudes toward rehabilitating inmates. Since the variable perceived OT has a role in prison settings was uniquely associated with support for rehabilitating inmates, fostering the development of supportive attitudes toward rehabilitating inmates may be achieved through the following avenues: (1) educate students about roles of OT in prison setting by incorporating specific topics related to the criminal justice system in the OT academic curriculum as an emerging area of practice, (2) invite occupational therapists working in prison settings as guest lecturers to introduce their role to the students, and (3) create fieldwork experiences for students working in prison settings in which an occupational therapist supervises them.

We have attempted to reach out to program directors of other OT programs in Alabama, United States and requested them to distribute the invitation e-mail (questionnaire) to their current students. However, based on the internet protocol addresses of the student respondents in survey results, it seems none of them came from other programs. This study is potentially limited by the convenience sample of OT students from one university in Alabama which limits the generalizability of the findings to OT students with similar characteristics. In addition, key explanatory and response variables were from self-reported attitudes measures; hence, there is the possibility that respondents might have been biased in their responses.

## Figures and Tables

**Table 1.** Frequency, means and standard deviations for the respondents’ support for rehabilitation (n=128)

Item		Disagree (%)	Neutral (%)	Agree (%)	Mean±standard deviation
1.	All rehabilitation programs have done is to allow criminals who deserve to be punished to get off easily.	67.2	21.1	11.7	5.27±1.50
2.^a)^	Rehabilitating a criminal is just as important as making a criminal pay for his or her crime.	15.6	14.8	69.5	5.25±1.61
3.^a)^	The most effective and humane cure to the crime problem in America is to make a strong effort to rehabilitate offenders.	11.7	13.3	75.0	5.25±1.54
4.	The only way to reduce crime in our society is to punish criminals, not try to rehabilitate them.	68.7	8.6	22.7	5.09±1.74
5.	We should stop viewing criminals as victims of society who deserve to be rehabilitated and start paying more attention to the victims of these criminals.	30.5	36.7	32.8	4.05±1.47
6.^a)^	I would support expanding the rehabilitation programs with criminals that are now being undertaken in our prisons.	10.2	8.6	81.2	5.49±1.41
7.^a)^	One of the reasons why rehabilitation programs often fail with prisoners is because they are under-funded; if enough money were available, these programs would work.	17.2	20.3	62.5	4.84±1.46
8.	The rehabilitation of adult criminals just does not work.	70.3	10.9	18.8	5.16±1.68
9.	The rehabilitation of prisoners has proven to be a failure.	65.6	18.0	16.4	5.16±1.63

a) Responses to items 2, 3, 6, and 7 had been recoded in data analysis, this was reflected in the mean scores.

**Table 2.** Background characteristics and variable responses of the respondents (n=128)

Variable	Response	No. of respondents	Rehabilitation Orientation Scale score	P-value
Gender	Female	117	5.15±1.12	0.006
	Male	11	4.13±1.43	
Race	White	115	5.09±1.19	0.45
	Non-White	13	4.83±1.03	
Year in the occupational therapy program	Yr1	54	5.09±1.37	0.84
	Yr2	48	4.99±1.11	
	Yr3	26	5.15±0.84	
Perceived occupational therapy has a role in the prison system	Yes	117	5.17±1.15	0.001
	No	11	3.94±0.87	
Had exposure to a therapist working in the prison setting	Yes	18	5.63±0.63	0.001
	No	110	4.97±1.22	
Knowing someone who has been incarcerated	Yes	66	4.87±1.27	0.08
	No	62	5.24±1.05	

Values are presented as mean±standard deviation, unless otherwise stated.

**Table 3.** Bivariable and multivariable linear regression analyses examining factors associated with scores of Rehabilitation Orientation Scale

Explanatory variable	Bivariable analysis			Multivariable analysis		
	B	SE	P-value	B	SE	P-value
Consideration of working in prison settings	0.22	0.07	0.001	0.18	0.07	0.006
Perceived occupational therapy has a role in prison settings	1.23	0.36	0.001	0.92	0.36	0.01
Knowing someone who has been incarcerated	-0.36	0.21	0.08	-0.37	0.20	0.06
Gender	1.02	0.36	0.006	0.61	0.36	0.09
Had exposure to a therapist working in the prison setting	0.66	0.29	0.03			

B, unstandardized regression coefficient; SE, standard error.

## Supplementary materials

Supplement 1. Data files are available from https://doi.org/10.7910/DVN/XVUMRK.

Supplement 2. Audio recording of the abstract.
